# Living in the Restoration Public Housing after the Great East Japan Earthquake Correlates with Lower Subjective Well-Being of Older Adults

**DOI:** 10.3390/ijerph16152696

**Published:** 2019-07-28

**Authors:** Nobuaki Moriyama, Hajime Iwasa, Masaharu Tsubokura, Yujiro Kuroda, Seiji Yasumura

**Affiliations:** 1 Department of Public Health, Fukushima Medical University School of Medicine, Fukushima, Fukushima Prefecture 960-1295, Japan; 2 Center for Integrated Science and Humanities, Fukushima Medical University, Fukushima 960-1247, Japan; 3 National Institute of Advanced Industrial Science and Technology, Tsukuba, Ibaraki Prefecture 305-8560, Japan

**Keywords:** subjective well-being, social capital, emergency evacuation, Great East Japan Earthquake, older adults

## Abstract

We aimed to (1) describe the subjective well-being (SWB) of older residents in Fukushima Prefecture seven years and seven months after the Great East Japan Earthquake (GEJE) and examine the effect of relocation to the restoration public housing (RPH) on SWB, social capital, and health indicators; and (2) investigate the association between social capital and SWB. Questionnaires were administered to collect data of both RPH and non-RPH residents (≥65 years). Respondents’ SWB was collected via the Japanese version of the World Health Organization Five Well-Being Index. Additionally, residents’ social capital (trust, reciprocity, and participation), physical activity level, social network, functional health, history of chronic disease, and demographic data were collected. We analyzed 101 responses (valid response rate: approximately 34%) from RPH and 158 (53%) from non-RPH residents. SWB was lower in RPH compared to non-RPH residents but not statistically significant. Older RPH residents may demonstrate lower social capital and health indicators after the GEJE. Mistrust was found to be positively associated with low SWB in RPH residents. Future studies should examine the effectiveness of support for enhancing the trust of older RPH residents regarding, for example, the involvement of scientists—including medical professionals—in risk communications in promoting SWB.

## 1. Introduction

Subjective well-being (SWB) refers to thoughts and feelings associated with one’s own well-being [1] based on the presence of positive emotions and moods (e.g., contentment, happiness), the absence of negative emotions (e.g., depression, anxiety), satisfaction with life fulfillment, and positive functioning [2,3]. Well-being has several health benefits, such as improved prognosis and survival of people aged 75 years and older [4], as well as better immune responses and tolerance to pain [5].

On 11 March 2011, the Great East Japan Earthquake (GEJE) and the subsequent tsunami caused the reactors at the Fukushima Daiichi Nuclear Power Plant to explode, which resulted in radioactive contamination of the surrounding areas. As a result, 19,689 people lost their lives, and over 2500 were still officially reported as missing as of 1 March 2019 [6]. The disaster also caused marked deterioration in the measurements of clinical parameters related to lifestyle diseases such as systolic and diastolic blood pressure, blood glucose levels, and triglyceride levels in Fukushima Prefecture [7].

Because of the risk of radiation exposure, the Japanese government restricted the entry into populated areas that were contaminated, thereby forcing the long-term evacuation of citizens from their homes [8]. In addition, anxiety about contamination was reported to cause deterioration of SWB of the residents in the town of Marumori, Miyagi Prefecture, which is located 50 km northwest of the power plant [9]. Since SWB contributes to general health in older people, deterioration of SWB after this great disaster is a public health concern.

With the ordered evacuation, it took a long time for many residents to return to their original homes or to find other permanent places to live, and they underwent relocation several times. First, many residents were evacuated to the designated shelter. Then, most of them moved to relatives’ houses or housings of municipalities, which were ready to receive them until temporary housing in Fukushima Prefecture was ready. The number of Fukushima residents who were evacuated reached approximately 164,000 in April 2011 [10]. In July 2011, the Prefectural Government of Fukushima was able to receive the first residents in temporary housings. As the evacuation order was gradually lifted, temporary housing residents were asked to vacate the accommodations. For those whose areas of former residence were designated as restricted areas and those who had not yet decided where to live, the Prefectural Government of Fukushima built housing complexes called restoration public housing (RPH) in 15 municipalities within Fukushima Prefecture. On September 2014, RPH began receiving residents. It was reported that RPH residents experienced relocation three to eight times until finally settling there (median: four times) [11].

Regarding the impact of evacuation/relocation in older people after the GEJE, decreased physical activity levels [12] and mental stress [13] were reported. Furthermore, relocation and environmental change can cause a loss of social capital. Social capital has been identified as an important social determinant of health, and it is formally defined as the resources that individuals can access via their social network [14]. Since social capital has been reported to be significantly associated with lower risks for depressive symptoms in community-dwelling older Japanese people [15], deterioration of social capital should be prevented.

As of July 2018, there were approximately 4000 households in RPH (exact number unavailable), occupying approximately 85% of the capacity of all constructed complexes. Most RPH residents could neither return to their original homes nor build new houses in places other than the original hometown. Because RPH residents were forced to live in unfamiliar situations and neighborhoods, with many of them having been separated from their families and communities [16], it is possible that they had low social capital. In addition, the community of RPH in Fukushima Prefecture had a high proportion of older people living alone (45% according to the Government of Fukushima Prefecture). This situation among the aging society is common in the affected areas of the GEJE [17]. Older RPH residents might have experienced difficulty in asking for support from neighborhoods, which may have caused a vicious circle of low SWB.

Although a previous study examined the effect of residential relocations after the GEJE on social capital [18], little is known about older RPH residents’ social capital, or whether their social capital was associated with their SWB during the reconstruction phase of the GEJE. Thus, in the present study, we aimed to (1) describe the SWB of older residents in Fukushima Prefecture seven years and seven months after the GEJE and examine the effect of relocation to RPH on their SWB, social capital, and health indicators; and (2) investigate the association between social capital and SWB for them. We hypothesized that (1) SWB, social capital, and health indicators would be more deteriorated in residents living in RPH compared to non-RPH residents, and that (2) social capital would be associated with SWB in RPH residents. The findings of this study could provide relief workers or health care providers in similarly affected areas with some guidance for the improvement of SWB and health outcomes in local older citizens, especially those who experienced evacuation/relocation in the medium- to long-term after GEJE.

## 2. Materials and Methods

### 2.1. Study Design

This study used a cross-sectional design. The report of the study follows the recommendations of the Strengthening the Reporting of Observational Studies in Epidemiology (STROBE) [19].

### 2.2. Setting

The study sites included the cities of Fukushima, Koriyama, and Iwaki, which are the three largest cities according to the population in Fukushima Prefecture; all have a population of 290,000 to 340,000 people. As of February 2019, the distribution of the capacity of households residing in RPH was 475 in Fukushima City, 570 in Koriyama City, and 1672 in Iwaki City.

### 2.3. Participants

This survey targeted residents of Fukushima Prefecture aged 65 years or older. We sent questionnaires to 600 households living in RPH (200 for each city; RPH group) and to 300 households in other types of housing (100 for each city; non-RPH group). The selection was based on a two-stage stratified random sampling (stage one corresponding to the municipality, stage two corresponding to individuals). The survey was administered as an anonymous, self-reporting postal questionnaire. The survey period was from 30 October to 27 November 2018. To select subjects living in non-RPH housing, we used the Basic Resident Register, which comprises documents concerning proof of residence stored at the municipality office. Regarding RPH households, we could not use the same register to sample RPH residents because many of them have maintained their resident registration in their original municipalities. Therefore, to choose the RPH sample, first, we randomly selected the units of housing complexes. According to the latest national census in 2015 [20], 50% of households have at least one older person. Thus, we posted the questionnaire to 600 RPH households, which we expected to have approximately 300 RPH residents over 65 years old. We attached a letter of request asking the recipient to give the questionnaire to the person with the earliest birthday who is at least 65 years old or older.

The sample size for the multiple regression analysis was previously estimated with nine independent variables. As a previous study suggested that the number of participants per independent variable should be 10 or greater [21], at least 90 subjects were necessary per group categorized by housing type in case we performed stratified analysis. Assuming that the rate of valid response was 50%, the sample for questionnaire distribution was estimated to be 180 from both RPH residents and non-RPH residents.

In total, 311 people responded to the questionnaire. Of them, 122 were from the RPH group, and 189 were from the non-RPH group. The response rate of non-RPH residents was 63.0%. The approximate response rate of RPH residents was estimated at 40%. A total of 21 RPH residents and 31 non-RPH residents were excluded for providing incomplete or inadequate answers; we analyzed the remaining 101 RPH and 158 non-RPH responses (total: 259).

### 2.4. Dependent Variable

The dependent variable was the participant’s SWB. To assess SWB, the Japanese version of the World Health Organization Five Well-being Index (WHO-5-J) was administered. The WHO-5-J is useful for measuring mental health in older, community-dwelling Japanese adults [22] and consists of the following five items to measure participants’ SWB over a two-week period: (1) felt cheerful and in good spirits, (2) felt calm and relaxed, (3) felt active and vigorous, (4) woke up feeling fresh and rested, and (5) daily life filled with things that interest me. The response to each item was rated on a six-point Likert scale ranging from 0 to 5 for a possible maximum score of 25 points. Higher scores indicated better SWB.

### 2.5. Key Independent Variable: Social Capital

As for measuring social capital, a previous study by Harpham provided evidence for the importance of distinguishing cognitive components (cognitive social capital) from structural components (structural social capital). Cognitive social capital refers to what people feel (e.g., trust in others, reciprocity between individuals), whereas structural social capital refers to what people do (e.g., participation in associations) [23].

Respondents’ perceived trust of others and reciprocity were employed to assess cognitive social capital, and social participation was used to evaluate structural social capital. Respondents’ trust as a measure of cognitive social capital was assessed through the following question: “Generally speaking, would you say that most people in your community can be trusted?” The question was rated in terms of five responses: 1 = can be trusted, 2 = can be somewhat trusted, 3 = neither, 4 = cannot be somewhat trusted, or 5 = cannot be trusted. The second item concerned reciprocity: “Would you say that most people in your community are willing to help each other?” Participants could answer: 1 = think so, 2 = somewhat think so, 3 = neither, 4 = somewhat don’t think so, or 5 = don’t think so. Responses were dichotomized into the first three alternatives and the two latter alternatives for the analysis; mistrust and lack of reciprocity corresponded to responses 4 and 5 for both questions, in accordance with the criteria used in previous studies [24,25].

To measure social participation, respondents were asked whether they belonged to the following organizations: “territorial organizations”, “exercise or sports groups”, “volunteer activity groups”, “hobby and lifelong studies groups”, “religious groups”, or “political organizations”. Responses were categorized into three groups—“absence”, “one or two”, and “more than three”—according to the number of activity types they belonged to out of the six types of organizations.

### 2.6. Covariates

We considered physical activity level, social network, functional health, history of chronic disease, and demographic information (age, sex, household structure) as covariates for the multivariate analysis to examine the association between SWB and social capital.

Physical activity level was evaluated using the Physical Activity Questionnaire for Elderly Japanese (PAQ-EJ) [26]. PAQ-EJ scores were converted to metabolic equivalent of task (MET) hours per week (MET h/week). Respondents were classified into two groups: those with a physical activity level greater than or equal to the reference value of 10 MET h/week proposed by the Japan Ministry of Health, Labour and Welfare [27], and those with a lower level of physical activity.

The social network was evaluated as a distinguished variable to represent social capital [28]. The number of friends was measured to indicate the scale of the social network. We determined the number of friends using the following question: “How many friends did you meet in the previous month?” Participants could answer: 1 = zero, 2 = one to two, 3 = three to five, 4 = six to nine, or 5 = ten or more. Responses were dichotomized with the first three alternatives corresponding to a small number of friends and the two latter alternatives corresponding to a large number of friends for the analysis.

A history of chronic disease (hypertension, diabetes, cerebral stroke, and heart disease) was evaluated using the following question: “Have you ever been diagnosed or received treatment for the specified disease?” Respondents chose one answer from “no”, “regularly go to the hospital”, “cured”, and “neglect”. These answers were dichotomized into “presence” based on whether respondents answered “regularly go to hospital” or “neglect” for at least one item or “absence” if they answered no” or “cured”.

The functional health was evaluated using the Tokyo Metropolitan Institute of Gerontology Index of Competence (TMIG-IC) [29]. The response to each item was designed simply as “yes” (able to do) or “no” (unable) and scored 1 for “yes” and 0 for “no”. The total score was the sum total of 13 items, in which scores ranging from 0 to 13 indicated functional capacity in instrumental activities of daily living (e.g., being able to shop, prepare meals), intellectual activities (e.g., reading newspapers, books), and social roles (e.g., visiting friends) and were categorized as follows: independence (13 points) or dependence (≤12 points) [30].

We also obtained the subjects’ demographic information (age, sex, household structure). Respondents’ household structure was evaluated by the question, “Please choose the structure of your household”. The responses were “living alone”, “couple”, “nuclear family”, “three-generation family”, and “other”. The responses were categorized into three groups: “living alone”, “couple/nuclear family/other”, and “three-generation family”.

### 2.7. Statistical Methods

First, we examined the differences in SWB, each type of social capital, and other variables between groups divided according to the type of residence. To compare the variables between groups, a chi-square test or a t-test was used.

Second, we examined the association between SWB and each type of social capital. Univariate analysis (t-test or the Pearson product-moment correlation coefficient) was performed between SWB and all measured variables. We then performed multiple regression analysis. The model included scores of the WHO-5-J (representative of SWB) as a dependent variable and each type of social capital (trust, reciprocity, and participation) as key independent variables. In addition, age, sex, chronic disease history, household structure, and other variables that were found to be associated with SWB in the univariate analysis were included as covariates. Thus, three regression models were constructed, in which the independent variable was each of the three types of social capital. To determine the effect of relocation in RPH on the association between SWB and social capital, an interaction term for a type of residence and social capital was entered in the multiple regression models. In addition, multiple regression analysis stratified by type of residence was also performed. The level of significance for all analyses was set at *p* < 0.01. All data were analyzed using IBM SPSS Statistics for Windows, version 21 (IBM Corporation, Armonk, NY, USA).

### 2.8. Ethical Issues

The study was approved by the Fukushima Medical University’s Ethics Committee (Approval number: 30104) and conducted in accordance with the ethical principles of the Declaration of Helsinki. We considered a returned questionnaire to be a participant’s consent to the objective of the study and their voluntary participation in it.

## 3. Results

### 3.1. Characteristics of Participants

Participant characteristics by groups divided according to the type of residence (RPH/non-RPH) are summarized in Table 1. Significantly higher numbers of participants with lower functional health and who were living alone were found in the RPH group compared to the non-RPH group (Table 1).

### 3.2. Status of SWB, Social Capital, and Health Indicators in Subjects between Groups among Groups

**Figure 1:**
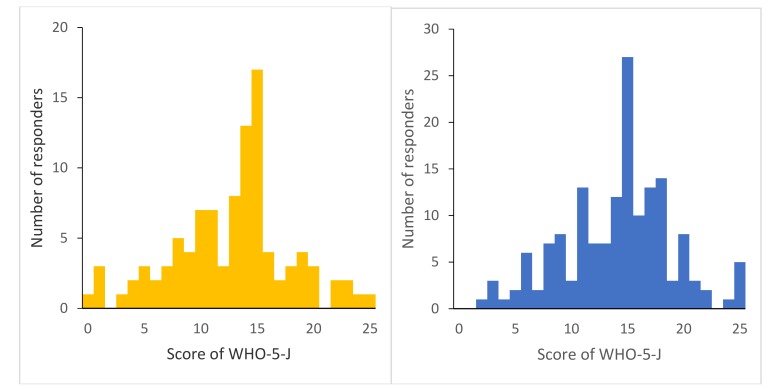
Distribution of the score of the Japanese version of the World Health Organization Five Well-being Index (WHO-5-J). **Left**: RPH group (n = 101), **Right**: non-RPH group (n = 158).

The mean score of the WHO-5-J as a representative variable of SWB in this study was 13.59 ± 5.00. Regarding comparison of WHO-5-J between groups according to the type of residence, the WHO-5-J score was 12.76 ± 5.18 in the RPH group and 14.11 ± 4.82 in the non-RPH group, respectively. The score was lower in the RPH group compared to the non-RPH group but not statistically significant (*p* = 0.03, 99% CI: −2.993, 0.290; Table 2). The distribution of the WHO-5-J score is shown in the figure below. The kurtosis and the skewness were 0.160 and −0.203 in the RPH group and −0.001 and −0.180 in the non-RPH group, respectively (Figure 1).

In the comparison of participants’ social capital as key independent variables, significantly higher numbers of participants with mistrust and lack of reciprocity were observed in the RPH group than in the non-RPH group (Table 2). Less participation was observed in the non-RPH group compared to the RPH group, although the difference was not statistically significant (Table 2).

### 3.3. Association between SWB and Social Capital

The results of univariate analysis regarding the association between SWB and social capital suggested that mistrust and low participation were negatively associated with SWB (Table 3). Regarding the association between SWB and the covariates, being physically inactive, low social network, and being functionally dependent were negatively associated with SWB. The correlation coefficient between the WHO-5-J score and age was −0.22 (*p* = 0.728), indicating no statistical significance (the data table is not shown).

From the results of the univariate analysis, the multiple regression analysis included physical activity level, social network, and functional health, as well as age, gender, chronic disease history, and household structure (the data table is not shown). The result of multiple regression analysis performed on the data of both the RPH and non-RPH groups indicated that mistrust was negatively associated with SWB (standardized partial regression coefficient; β = −0.225, *p* < 0.01). However, lack of reciprocity (β = −0.089, *p* = 0.24) and less participation (β = −0.134, *p* = 0.47) were not significantly associated with SWB. Regarding the interaction for a type of residence and social capital, no significant interactions were observed between mistrust × RPH residence (β = −0.061, *p* = 0.42), lack of reciprocity × RPH residence (β = 0.040, *p* = 0.61), and low participation × RPH residence (β = −0.064, *p* = 0.33).

The stratified model of the multivariate analysis indicated that mistrust (β = −0.293, *p* < 0.01) was negatively associated with SWB for the RPH group; however, lack of reciprocity (β = 0.077, *p* = 0.43) and less participation (β = −0.238, *p* = 0.02) were not significantly associated with low SWB (Table 4). In the model for the non-RPH group, mistrust (β = −0.200, *p* < 0.01) was associated with low SWB, but lack of reciprocity (β = −0.125, *p* = 0.10) and less social participation (β = −0.038, *p* = 0.64) were not associated with low SWB (Table 5).

## 4. Discussion

Local citizens who were ordered by the government to evacuate after the GEJE suffered various social impacts on their health. This study examined the SWB and the social capital of older RPH residents and the association between the two by comparing them with those of residents from other types of housings. To our knowledge, this was the first study that attempted to clarify the impact of relocation and environmental change on SWB and the associated factors involved in the deterioration of SWB in RPH residents with a medium- to long-term perspective.

Regarding the distribution of the WHO-5-J scores, a previous study that provided normative data of the scores in community-dwelling older Japanese adults reported that the kurtosis and the skewness were −0.23 and −0.57 in men and −0.25 and −0.51 in women, respectively, and the mode was 20 in both men and women [31]. Compared with the previous normative data, the distribution of the score in this study cohort was slightly negatively skewed and leptokurtic. It is possible that, compared with normative Japanese data, the peak of distribution shifted from high scores to medium scores, which would mean that many older residents in Fukushima Prefecture after the GEJE had deteriorated SWB compared to older adults living in other places in Japan. This result suggested that, in the aftermath of the GEJE, the experience of the disaster impacted local older people’s mental health, and evacuation/relocation seemed to amplify the impact.

On the other hand, the difference in the WHO-5-J scores between the RPH and the non-RPH groups was not significant (1.35). The cohort study involving people living in Fukushima Prefecture after the GEJE [32] indicated that the percentage of participants in the evacuation zone specified by the government aged 16 and older who are suspected to have affective or anxiety disorders decreased continuously from fiscal year (FY) 2012 to FY 2014; however, no significant change was observed in the next three years [33]. As shown in this report, it is possible that, although the mental health of evacuees in Fukushima initially improved after the disaster, it remains deteriorated compared to that of people living in the normal settings. In addition, the average score of the above-mentioned normative data [31] is 16.5 among men and 16.3 among women. This result shows that residents in Fukushima Prefecture suffer from deteriorated mental health even seven years after the disaster and regardless of relocation. Therefore, it is necessary to provide continuous mental health care to older residents.

From the result regarding the comparison of parameters other than SWB between the RPH and the non-RPH groups shown in Table 1 and Table 2, lower cognitive social capital represented by trust and reciprocity, worse functional health, and higher incidence of living alone were observed in the RPH residents. In addition, higher prevalence of history of chronic disease was observed in the RPH residents, although the difference was not significant. On the other hand, structural social capital represented by participation was slightly higher but not significant in the RPH groups. All RPH residents had relocated to unfamiliar places in recent years. In addition, as a specific mental health consequence following the GEJE, self-stigma has been reported to be produced by public stigma related to radiation exposure among evacuees [34]. Such a background may have affected the lower cognitive social capital in RPH residents. The results showed that the social network represented by the number of friends was similar between the RPH and the non-RPH groups. This may be explained by the fact that some facilities in RPH could provide an opportunity to expand the social network; for example, each RPH complex has an assembly hall for residents to hold events and interact with each other. The deteriorated functional health may have resulted from the impaired physical performance in temporary housing residents, as previously reported [35]. Regarding the presence of a history of chronic disease, previous studies suggested that evacuation following the GEJE was associated with increased hypertension [36], metabolic syndrome [37], and diabetes [38]. Although it was reported that the risk of hypertension and diabetes was not significantly different between evacuees and non-evacuees [39], the onset of chronic diseases could increase in the RPH group in the post-disaster years.

In summary, older residents in RPH reported a lower SWB (not statistically significant), limited cognitive social capital, and deteriorated functional health. As mentioned above, RPH residents have moved several times. A previous study also suggested that survivors who experienced multiple relocations after the GEJE may have been subjected to cumulative stress [40]. Furthermore, daily life in an unfamiliar environment may have made it difficult to manage chronic health issues and the creation of cognitive social capital. As the situation surrounding RPH may make it challenging to provide mutual aid among residents, official support or support from outsiders should be further provided to RPH residents.

Univariate analysis of the association between SWB and measured variables found that poor status of physical activity, social capital (trust, participation), social network (number of friends), functional health, and subjective health were associated with low SWB. This result is consistent with previous findings, which reported an association between well-being and physical activity [41], social network [42,43], subjective health, and functional health [44] Thus, the responders of this study seemed to be affected by the same factors as in normal settings, and it was appropriate to perform multivariate analysis with adjusting variables other than social capital.

In the multivariate analysis of both the RPH residents and the non-RPH residents, an association with SWB was found only for trust after adjusting for the covariate. This result is consistent with a previous study reporting that trust resources contributed to the SWB of adults living in affected areas six months after the GEJE [45]. In the multiple regression analysis stratified by type of residence, mistrust was found to be associated with low SWB in RPH residents after adjusting for covariates. Less participation tended to be associated with low SWB, although this association was not statistically significant. This tendency was supported by a previous report that the lack of social participation was significantly associated with psychological distress among evacuees after the GEJE [46]. However, reciprocity was negatively associated with SWB in RPH residents, although this association was not significant. The difference in direction in the association between SWB and social capital (trust and reciprocity) may be attributed to their different natures. A previous study that focused on Japanese-American older adults reported that those who had strong norms toward reciprocity were more depressed, showed more symptoms of aging, and had lower levels of psychological well-being than those who did not have strong reciprocity norms [47]. The author called this mechanism a “reciprocity-burden”, in reference to reciprocating the help provided earlier in life despite the impossibility of accomplishing the reciprocity norm due to age-related life challenges. Thus, reciprocity may not always have a positive effect on SWB. In the case of the present study, residents in RPH severely suffered because of the GEJE and the consequent radiation accident; however, they also received support from various resources to reconstruct their lives. It is possible that some responders among RPH residents felt the “reciprocity-burden” and subsequently failed to improve SWB.

Regarding the effect of covariates, being female, having a lower social network, and poorer functional health had stronger effects on low SWB in the non-RPH group compared with the RPH group. This result suggests that SWB of local older residents living in their own residences is affected by the same previously reported predictors. Moreover, a previous study reported that women are more vulnerable during disasters compared to men because of genetic, psychological, physiological, and social factors [48], and that women living in affected areas after the GEJE were more likely to suffer from mental health problems [40]. On the other hand, residents of RPH have moved several times, which was more stressful for them. It is possible that the effect of covariates on SWB in RPH residents was relatively weakened. Regarding the sex difference, being male is an indicator of adverse effects of involuntary relocation on physical and mental health [49]. Thus, the effect of sex seen in the non-RPH group disappeared because of multicomplex circumstances.

The strength of this study is that the participants were randomly chosen from the population to minimize selection bias of the attained data. Furthermore, the study was conducted in three large municipalities, thus the respondents were representative of an entire target population. However, this study also has some limitations. First, because of the nature of the cross-sectional design, causal relationships between SWB and associated factors could not be established. Second, although previous studies have suggested that socioeconomic factors (e.g., income and employment status) are associated with SWB, no information was obtained from respondents about these factors. In the case of the Fukushima accident, evacuees after the nuclear accident have received a monthly compensation for mental anguish from Tokyo Electric Power Company Holdings Inc. (Tokyo, Japan), which owns the power plant, and this has been a cause of discrimination against the evacuees wherever they lived [50]. This issue should be treated delicately, and we assumed that it may have been difficult for the evacuees to discuss the effects of their financial situation on SWB. Therefore, we opted against collecting data on the participants’ financial situations. Thus, the multivariate analysis applied in this study failed to adjust for this factor, which may have affected the association between SWB and social capital. Third, data on respondents’ SWB before the disaster or relocation were not collected. Hence, it was hard to determine whether low SWB was due to experiencing the disaster or subsequent major life events (e.g., relocation), or if it was originally low. With information on pre-disaster SWB, it would be possible to more thoroughly discuss the impact of the disaster and relocation on SWB of older residents. Finally, the valid response rate from RPH residents could not be calculated and was assumed to be not high, which may have affected the generalizability of this study. Despite these limitations, this present study newly found that residence in RPH impacted a change in household structure and deficits in cognitive social capital in older adults. In addition, we also found that trust was positively associated with SWB in older RPH residents in the medium- to long-term after GEJE.

As the next step, a longitudinal study should be conducted to clarify the causal relationship between SWB and the social capital of evacuees after the GEJE. If our expectation regarding the direction in which social capital affects SWB in evacuees after the GEJE is clarified, we will be able to provide information to the people in charge of the evacuees on the necessity of maintaining their social capital. Previous studies have attempted to maintain a general level of trust among residents in the setting of post-GEJE. To build trust in older residents in Fukushima Prefecture after the GEJE, the involvement of scientists including medical professionals in risk communications is desirable [51]. It may also be an effective strategy for professionals to provide reliable scientific information through the media [52]. Further studies are needed to explore possible interventions to improve trust in older RPH residents and test their effectiveness.

## 5. Conclusions

Older people in Fukushima Prefecture may show a lower SWB after the GEJE, and the evacuation/relocation may be an accelerator of low SWB. Mistrust was associated with low SWB in RPH residents. Further studies are necessary to examine the causal relationship between SWB and social capital in older RPH residents, which can help in developing health promotion strategies for them.

## Figures and Tables

**Table 1:** The demographic characteristics of the two respondent groups.

		RPH	Non-RPH	Total	*p*
		n = 101 (%)	n = 158 (%)	n = 259 (%)	
Sex	Male	48 (47.5)	75 (47.5)	123 (47.5)	0.99
	Female	53 (52.5)	83 (52.5)	136 (52.5)	
Age	Mean (Standard deviation)	75.0 (7.6)	73.8 (6.6)	74.3 (7.0)	0.12
Physical activity level	Inactive	17 (16.8)	21 (13.3)	38 (14.7)	0.43
	Active	84 (83.2)	137 (86.7)	221 (85.3)	
Social network	Low	50 (49.5)	75 (47.5)	125 (48.3)	0.75
	High	51 (50.5)	83 (52.5)	134 (51.7)	
Functional health	Dependent	72 (71.3)	86 (54.4)	158 (61.0)	<0.01
	Independent	29 (28.7)	72 (45.6)	101 (39.0)	
Chronic disease history	Presence	79 (78.2)	105 (66.5)	184 (71.0)	0.04
	Absence	22 (21.8)	53 (33.5)	75 (29.0)	
Household structure	Living alone	57 (56.4)	20 (12.7)	77 (29.7)	<0.01
	Couple/nuclear family/other	43 (42.6)	125 (79.1)	168 (64.9)	
	Three-generation family	1 (1.0)	13 (8.2)	14 (5.4)	

RPH: restoration public houses; SWB: subjective well-being, WHO-5-J score.

**Table 2:** SWB and social capital of the two respondent groups.

		RPH	Non-RPH	Total	*p*-Value
		n = 101 (%)	n = 158 (%)	n = 259 (%)	
SWB	Mean (Standard deviation)	12.76 (5.18)	14.11 (4.82)	13.59 (5.00)	0.03
Trust	Mistrust	63 (62.4)	44 (27.8)	107 (41.3)	<0.01
	High	38 (37.6)	114 (72.2)	152 (58.7)	
Reciprocity	Lack of reciprocity	45 (44.6)	38 (24.1)	83 (32.0)	<0.01
	High	56 (55.4)	120 (75.9)	176 (68.0)	
Participation	Absence	42 (41.6)	69 (43.7)	111 (42.9)	0.40
	One or two	40 (39.6)	69 (43.7)	109 (42.1)	
	More than three	19 (18.8)	20 (12.7)	39 (15.1)	

RPH: restoration public houses; SWB: subjective well-being, score of WHO-5-J.

**Table 3:** The association between SWB and other variables (social capital and covariates).

		WHO-5-J Score	*p*	99% CI
		Mean (SD)		
Trust	Mistrust (n = 107)	11.58 (4.47)	<0.01	1.88, 4.96
	High (n = 152)	15.00 (4.88)		
Reciprocity	Lack of reciprocity (n = 83)	12.67 (4.85)	0.04	−0.37, 3.06
	High (n = 176)	14.02 (5.02)		
Participation	Absence (n = 111) ^a^	12.25 (5.34)	<0.01	b-a: −0.09, 3.77 c-a: 1.05, 6.39 c-b: −0.79, 4.56
	One to two (n = 109) ^b^	14.09 (4.11)		
	More than three (n = 39) ^c^	15.97 (5.20)		
Sex	Male (n = 123)	13.30 (4.69)	0.34	−1.83, 0.64
	Female (n = 136)	13.90 (5.32)		
Physical activity level	Inactive (n = 38)	9.89 (5.74)	<0.01	1.69, 6.96
	Active (n = 221)	14.22 (4.58)		
Social network	Low (n = 125)	11.97 (4.92)	<0.01	1.59, 4.66
	High (n = 134)	15.10 (4.60)		
Functional health	Dependent (n = 158)	11.28 (5.00)	<0.01	1.84, 4.87
	Independent (n = 101)	15.63 (4.28)		
Chronic disease history	Presence (n = 184)	13.43 (4.88)	0.43	−1.23, 2.32
	Absence (n = 75)	13.97 (5.29)		
Household structure	Living alone (n = 77) ^d^	13.47(4.73)	0.80	e-d: −1.94, 2.16 f-d: −3.35, 5.29 f-e: −3.28, 4.91
	Couple/nuclear family (n = 168) ^e^	13.57(5.20)		
	Three-generation family (n = 14) ^f^	14.43 (4.03)		

RPH: restoration public houses; SWB: subjective well-being.

**Table 4:** The association between social capital and low SWB (RPH group; n = 101).

	β	*p*	β	*p*	β	*p*
	(99% CI)		(99% CI)		(99% CI)	
Trust (for mistrust)	−0.293	<0.01	-	-	-	-
	(−0.535, −0.051)					
Reciprocity (for lack of reciprocity)		-	0.077	0.43	-	-
			(−0.175, 0.329)			
Participation (for less participation)		-	-	-	−0.238	0.02
					(−0.495, 0.020)	
Sex (for female)	−0.005	0.95	−0.043	0.67	−0.016	0.87
	(−0.258, 0.247)		(−0.307, 0.222)		(−0.274, 0.241)	
Age	0.084	0.38	0.119	0.24	0.123	0.21
	(−0.167, 0.336)		(−0.145, 0.384)		(−0.134, 0.379)	
Physical activity (for inactive)	−0.234	0.02	−0.271	<0.01	−0.205	0.04
	(−0.484, 0.016)		(−0.536, −0.005)		(−0.466, 0.056)	
Social network (for low)	−0.158	0.11	−0.155	0.14	−0.113	0.27
	(−0.417, 0.102)		(−0.431, 0.121)		(−0.385, 0.159)	
Functional health (for poor)	−0.151	0.14	−0.23	0.03	−0.196	0.06
	(−0.417, 0.116)		(−0.507, 0.048)		(−0.463, 0.072)	
Chromic disease history (for presence)	0	0.99	−0.044	0.65	−0.002	0.98
	(−0.239, 0.240)		(−0.296, 0.209)		(−0.248, 0.243)	
Household structure (for living alone)	−0.072	0.43	−0.107	0.26	−0.126	0.17
	(−0.309, 0.165)		(−0.354, 0.140)		(−0.367, 0.115)	

Multiple regression analysis adjusted for age, sex, physical activity level, functional health, number of friends, chronic disease history, and household structure.

**Table 5:** The association between social capital and low SWB (non-RPH group; n = 159).

	β	*p*	β	*p*	β	*p*
	(99% CI)		(99% CI)		(99% CI)	
Trust (for mistrust)	−0.2	<0.01	-	-	-	-
	(−0.395, −0.004)					
Reciprocity (for lack of reciprocity)		-	−0.125	0.1	-	-
			(−0.320, 0.070)			
Participation (for less participation)		-	-	-	−0.038	0.64
					(−0.255, 0.178)	
Sex (for female)	−0.149	0.05	−0.165	0.03	−0.163	0.03
	(−0.342, 0.044)		(−0.360, 0.029)		(−0.363, 0.037)	
Age	0.038	0.63	0.066	0.41	0.08	0.32
	(−0.169, 0.245)		(−0.141, 0.273)		(−0.128, 0.287)	
Physical activity (for inactive)	−0.188	0.02	−0.193	0.01	−0.185	0.02
	(−0.388, 0.012)		(−0.395, 0.010)		(−0.392, 0.022)	
Social network (for low)	−0.207	0.01	−0.211	0.01	−0.212	0.02
	(−0.420, 0.007)		(−0.428, 0.005)		(−0.444, 0.019)	
Functional health (for poor)	−0.202	0.01	−0.215	0.01	−0.222	<0.01
	(−0.416, 0.011)		(−0.431, 0.002)		(−0.440, −0.004)	
Chromic disease history (for presence)	0.06	0.42	0.049	0.51	0.045	0.58
	(−0.133, 0.253)		(−0.147, 0.245)		(−0.154, 0.243)	
Household structure (for living alone)	−0.038	0.6	−0.101	0.54	−0.068	0.36
	(−0.153, 0.228)		(−0.148, 0.239)		(−0.126, 0.262)	

Multiple regression analysis adjusted for age, sex, physical activity level, functional health, number of friends, chronic disease history, and household structure.
